# COVID-19 Pandemic: Two-year Experience and Response of a Teaching Hospital in Malaysia and the Effect on Postgraduate Orthopaedic Training

**DOI:** 10.5704/MOJ.2207.001

**Published:** 2022-07

**Authors:** RY Kow, KA Khalid, Z Zakaria, MS Awang

**Affiliations:** 1 Department of Orthopaedics, Traumatology and Rehabilitation, International Islamic University Malaysia, Kuantan, Malaysia; 2 Director’s Office, Sultan Ahmad Shah Medical Centre @IIUM, Kuantan, Malaysia

**Keywords:** COVID-19, guideline, outbreak, pandemic, public health

## Abstract

As the number of COVID-19-related infections and deaths increased exponentially in the during 2020, few countries were equipped to manage and curb this novel coronavirus. Initially there was no proven cure or vaccine to this novel virus (SARS-Cov-2), leaving the authorities with no choice but to impose quarantines at the short-term expense of their economies. As we gain more knowledge on this novel virus, the tried-and-tested method of selective testing of the symptomatic patients, used successfully in almost all infectious respiratory diseases, has been replaced with trace-and-test method, as most of the infected patients remained asymptomatic. In early 2021, the availability of vaccines provided a shed of light out from this pandemic. Nevertheless, we faced an enormous task in juggling between vaccination of the population, managing patients with COVID-19 infection as well as non-COVID-19 patients. Here, we share our experience and response in managing this healthcare crisis across a two-year period during the pandemic and we hope other centres can learn from what we went through and help them derive a protocol to navigate through a future pandemic.

## Introduction

Prior to the start of the third decade of the 21st century, a danger was lurking when most Malaysians were preparing to welcome the long-awaited year of 2020. It started as a series of pneumonia of unknown cause in Wuhan, China in December 2019, which then spiralled out of control to affect other countries and subsequently the whole world^1,2^. The culprit, a novel coronavirus, was noted to be different from the other coronaviruses that had caused SARS (SARS-CoV) and MERS (MERS-CoV)^2^. The viral sequence was shared on 10th January 2020 through the community online resource *virological.org*^3,4^. Two days later, another four genomes were deposited in the viral sequence database curated by the Global Initiative on Sharing All Influenza Data (GISAID)^4^. The virus was later labelled as Severe Acute Respiratory Syndrome coronavirus two (SARS-CoV-2)^5^. On 30th January 2020, the World Health Organization (WHO) announced the disease as a Public Health Emergency of International Concern (PHEIC), when confirmed cases were detected in United States, Europe, Middle East and Australia^5,6^. The status escalated to become a pandemic on 12th March 2020, when there were more than 110,000 confirmed cases from 114 countries^7^. As of 28th September 2021, there were 231,703,120 confirmed cases with 4,746,620 deaths globally^8^.

In Malaysia, the director-general of health informed on 25th January 2020 that there were four confirmed cases of COVID-19, with all four being Chinese nationals had close contact with ill patients in Singapore entering the country^8^. The spread of the disease was further catalysed by a single mass gathering in Sri Petaling, Selangor, on 27th February 2020 to 1st March 20209. Through the implementation of the Movement Control Order (MCO), which began on 18th March 2020, Malaysia managed to flatten the curve, with the number of daily new cases remaining in a plateau, ranging from 16 to 110 infected people, with no exponential increase in cases as seen in other countries^10^. Until 22nd May 2020, there were 7059 confirmed cases with 114 deaths in Malaysia^10^. The situation in Malaysia escalated in 2021, when, despite all healthcare facilities in Malaysia adhering to the latest guidelines issued by the Ministry of Health, some hospitals were hit worse by the disease, as the majority of the cases were detected in Selangor and Kuala Lumpur^8^. There were 2,198,235 confirmed cases of COVID-19 and 25,437 confirmed deaths reported in Malaysia on 28th September 2021, with majority of them being reported in May to September 2021^11.^

## Early Responses

Herein, we provide a narrative review of the experience and response of the Sultan Ahmad Shah Medical Centre (SASMEC @IIUM), a tertiary university hospital in Kuantan, Pahang, Malaysia.

In 2020, the only and main hospital gazetted for treatment of COVID-19 patients in Kuantan, Pahang, under the Malaysian Ministry of Health, was Hospital Tengku Ampuan Afzan (HTAA). The Sultan Ahmad Shah Medical Centre, SASMEC, the university hospital, acted as a support hospital where patients with traumatic injuries and non-COVID-19-related illnesses were transferred from HTAA to offload the burden there. In order to ensure the safety of our staffs and patients, as well as to establish a mechanism in handling potential patients, we set up an IIUMMC COVID-19 Task Force was set up, led by Prof Dr Ahmad Hafiz Zulkifly and Prof Dato’ Dr Mohamed Saufi Awang, the supported by clinicians, nursing matrons, microbiologists, pharmacists, non-clinical support staffs and administrative and finance officers of the hospital. Realising that COVID-19 was a disease spread through close contact and respiratory droplets, all staffs were reminded to maintain a social distance of at least one meter and to practice proper frequent hand washing etiquette. A workflow was introduced to guide healthcare workers (HCWs) in contact with COVID-19 positive patients or patients under investigation (PUI). With the increment of COVID-19 cases in March 2020, the risk of staffs with close contact to patients with COVID-19 was stratified as high, medium and low risk and the management of exposed staffs to confirmed COVID-19 cases was based on the stratified risk. With the introduction of this HCWs risk stratification system, the risk of local transmission within the hospital was kept to the minimum, while retaining an adequate number of working personnel to keep the hospital functioning well.

## Clinics and Operation Theater

As the MCO was imposed on 18th March 2020, we postponed all elective surgeries such as arthroplasty and arthroscopy cases were postponed. Surgeries were reserved for patients with life- or limb-threatening conditions, such as open fractures, infections and intracranial bleeds. This was to preserve the in-patient beds and manpower in anticipation of a sudden surge of patients, owing to the overflow from HTAA, as well as to reduce the potential risk for patient-to-healthcare worker transmission. Complete segregation of healthcare workers was implemented to avoid interpersonal transmission among the healthcare workers.

As normal outpatient clinic, with a huge number of patients waiting in the waiting hall, potentially serves as a petri dish for viral multiplication. Realising the risk, non-essential clinic follow-ups were reduced, such as those on long term follow-up or non-interventional management. Only a limited number of clinic patients requiring active interventions, such as those with fractures, active infections and acute spine diseases, were retained. Off-site continuation of medications was enforced to limit the number of patient contact, whereby the number of HCWs was reduced and divided into teams to minimise contact with each other.

In compliance with to the MCO, and to avoid crowding, online video conferences was adopted for postgraduate continuous medical education (CME) programmes. Thus, we were unable to cater for patient physical examination training, as it would be unjustifiable to request patients to volunteer for educational purpose during this critical situation. On the positive side, since most of the other local and foreign universities embraced online teaching, our students had the opportunity to join some of the CMEs from them.

## Outbreak within the Hospital

**Fig 1:**
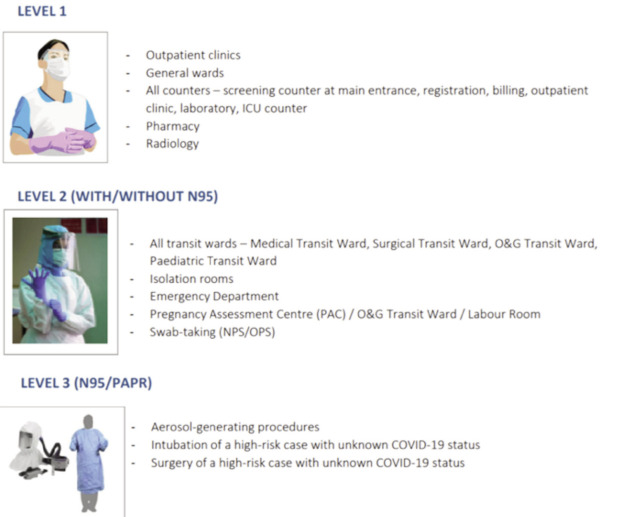
Schematic diagram of the level of personal protective equipment utilised in our centre.

As more knowledge and information about SARS-CoV-2 pathophysiology emerged, it became clear that traditional infection control and public health measures that had successfully contained and curbed SARS-CoV-1, which caused severe respiratory syndrome (SARS) in 2003, did not attain a similar effectiveness against SARS-CoV-212,13. This was because SARS-CoV-2 actively replicated in the upper respiratory tract, instead of the lower respiratory tract, enabling an infected pre- or pauci-symptomatic person to become contagious^14^. To make matters worse, a report by Arons *et al* highlighted the risk of COVID-19 silent transmission among HCWs, with more than 50% of the staff tested positive while being asymptomatic^15^. In light of the new discovery, re-strategisation by temporary cessation of decanting HTAA patients and the the imposition of mass screening of all HCWs and also inpatients were instituted. In other words, it meant the revision of the initial protocol on COVID-19 screening, from selective screening of those symptomatic to universal screening of all HCWs and patients at the hospital. As a result, a few of the HCWs and patients were found to be infected with COVID-19 and they were transferred to HTAA for further treatment. In consistence with other studies, during our universal screening of all HCWs and patients, a few asymptomatic patients, with no known exposure to those infected or suspected to be infected with COVID-19, were detected to be positive for SARS-CoV-2^16-17^. These patients had been admitted for non-respiratory diseases, such as spine surgical site infection and leukaemia. Since then, the admission protocol was altered in which all patients were required to undergo mandatory COVID-19 screening prior to admission and surgery. Those patients without life- or limb-threatening conditions were admitted to a surgical transit ward (STW) while waiting for the COVID-19 test results. Those with life- or limb-threatening conditions requiring immediate surgical intervention would undergo surgery with limited HCWs in level three personal protective equipment (PPE) in a COVID-19 designated negative pressure operating room (Fig. 1).

In view of the current situation experienced by our hospital, the management identified the main factor that had affected our service and HCWs as the lack of awareness and understanding on the COVID-19 disease. The first major step was to re-educate all our HCWs and supporting hospital services co-workers on the relevant basic knowledge, such as proper hand washing technique, selection of PPE and others. The standard operating procedures (SOPs) on how to handle surgical and medical patients was revised accordingly.

## A Period of Temporary Respite

During the period of MCO (Movement Control Order – 18th March 2020 to 3rd May 2020) and CMCO (Conditional Movement Control Order – 4th May 2020 to 9th June 2020), the number of COVID-19 cases were well under control^18^. With daily cases limited to single and double digits, HTAA was able to cope with the COVID-19 patients management, while we (SASMEC @IIUM) were able to alleviate their burden by managing non-COVID-19 patients. During this period, all elective surgeries were withheld or postponed^11^.

**Fig 2:**
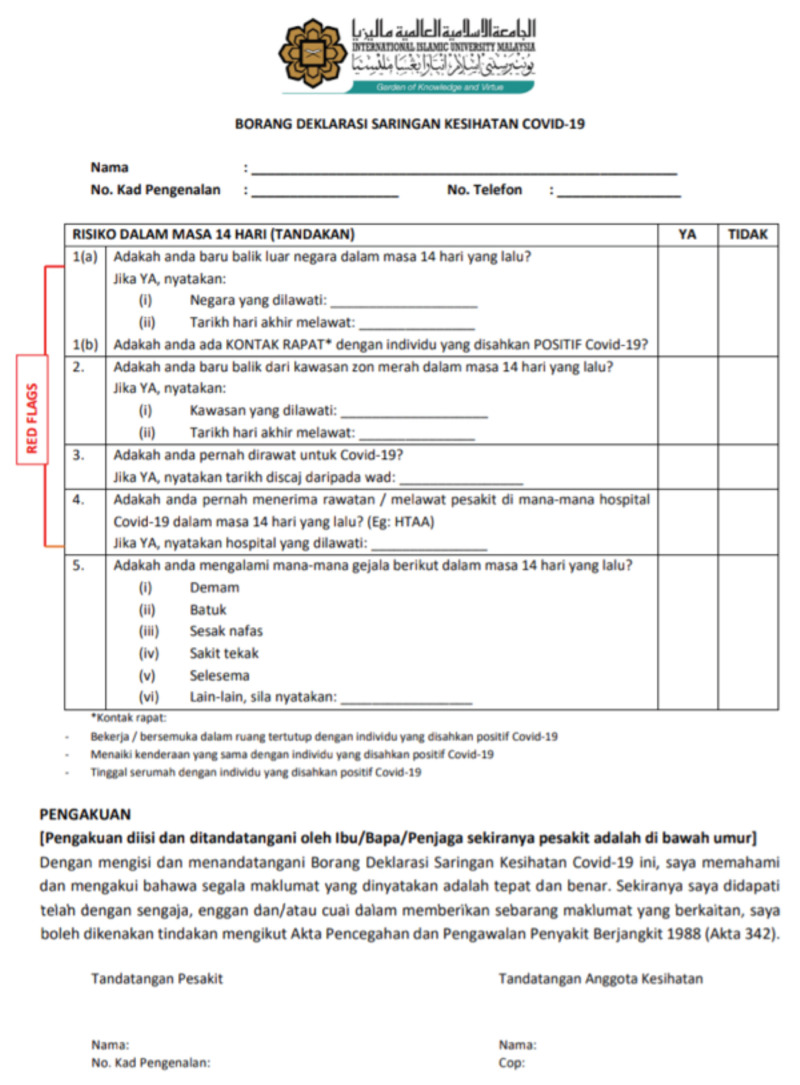
Declaration form to screen for COVID-19 during the early stage of the pandemic at Sultan Ahmad Shah Medical Centre @IIUM.

**Fig 3:**
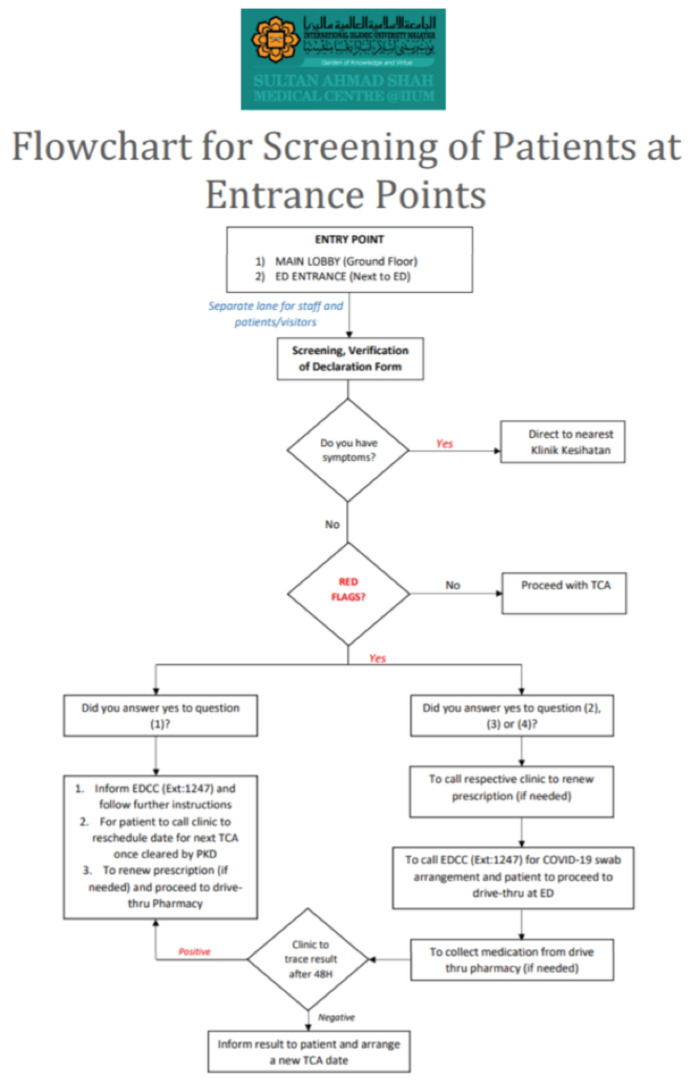
Flowchart for screening of patients at entrance points at Sultan Ahmad Shah Medical Centre @IIUM.

**Fig 4:**
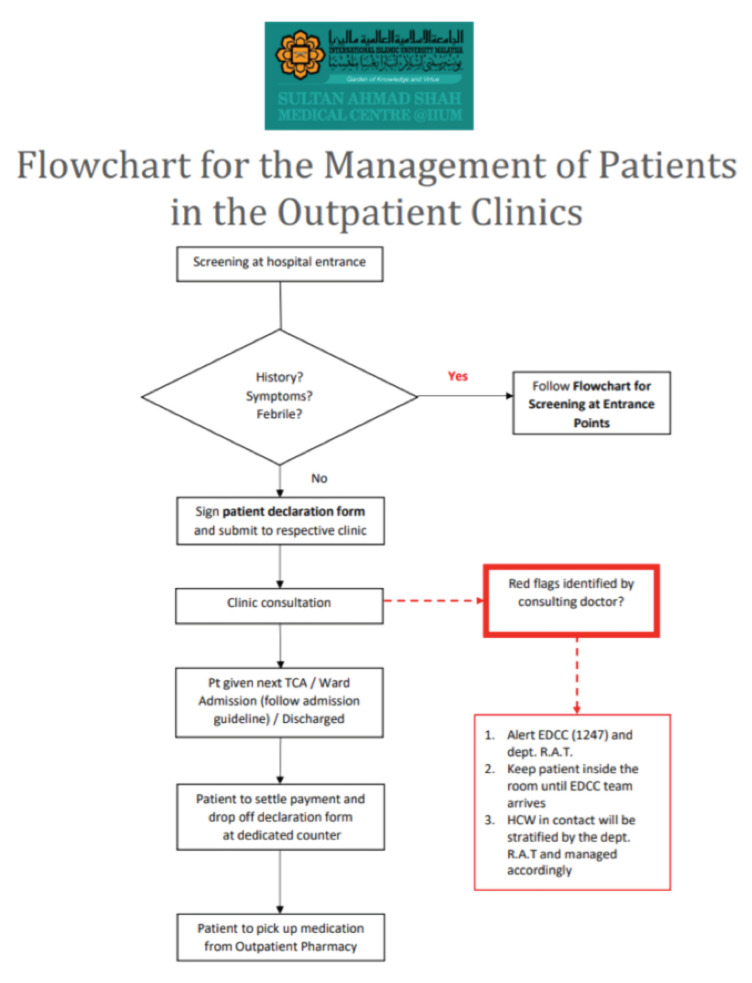
Flowchart for the management of patients in the outpatient clinics at Sultan Ahmad Shah Medical Centre @IIUM.

**Fig 5:**
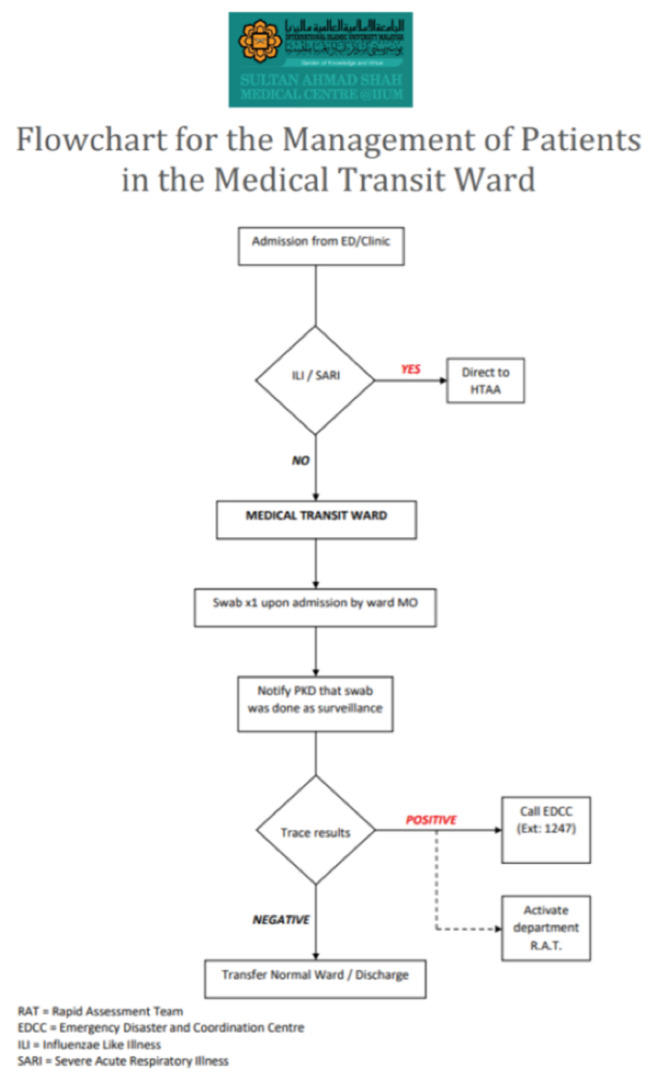
Flowchart for the management of patients in the medical transit ward at Sultan Ahmad Shah Medical Centre @IIUM.

**Fig 6:**
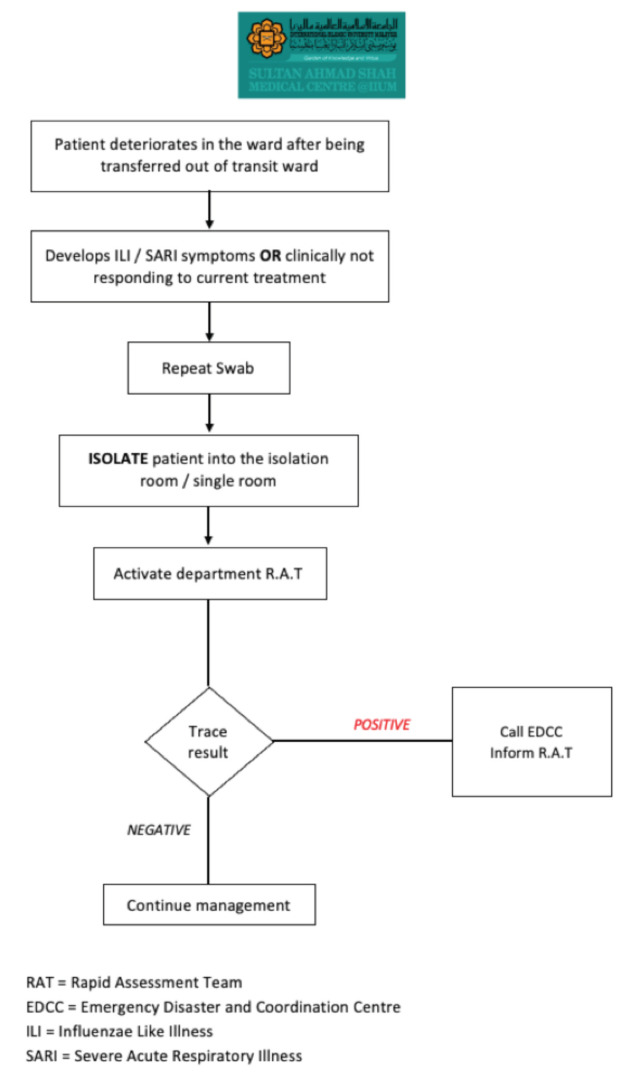
Flowchart for the management of deteriorating patients in the general ward at Sultan Ahmad Shah Medical Centre @IIUM.

**Fig 7:**
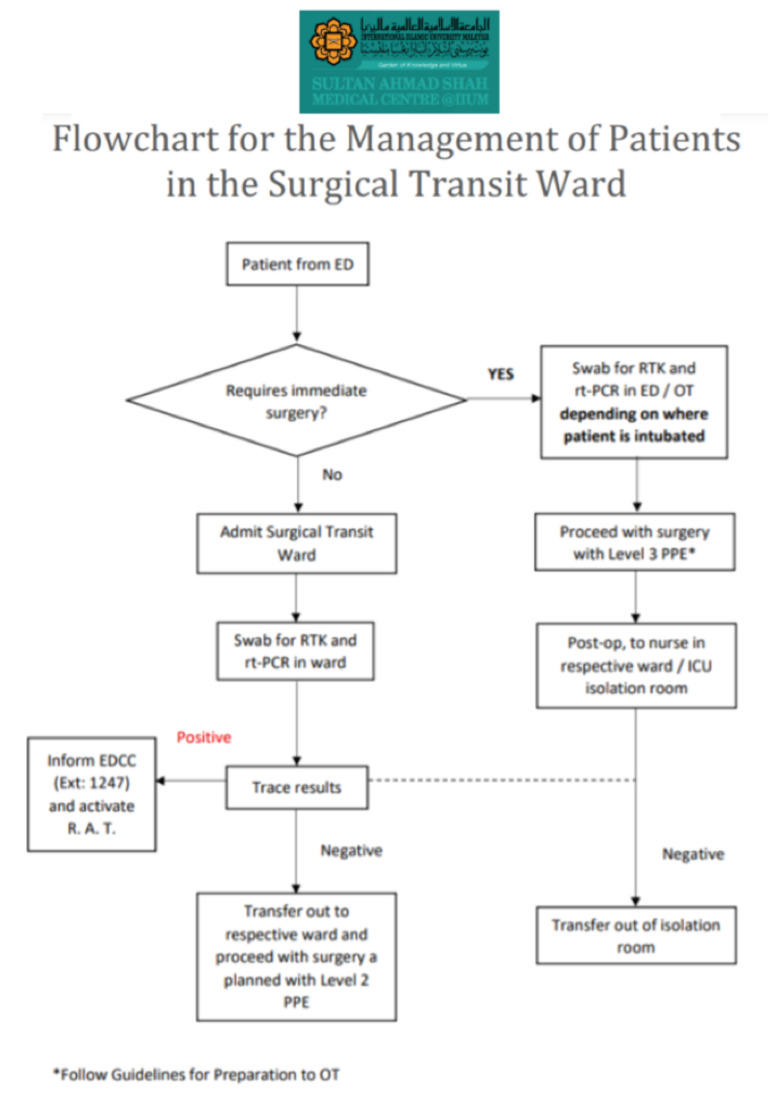
Flowchart for the management of patients in the surgical transit ward at Sultan Ahmad Shah Medical Centre @IIUM.

**Fig 8:**
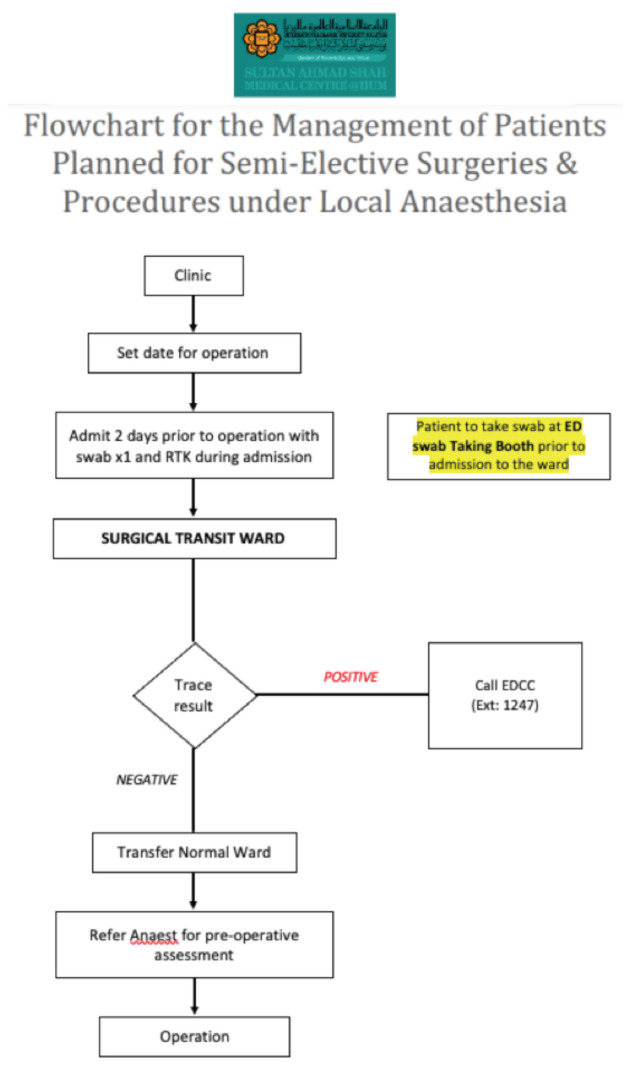
Flowchart for the management of patients planned for semi-elective surgeries & procedures under local anaesthesia at Sultan Ahmad Shah Medical Centre @IIUM.

**Fig 9:**
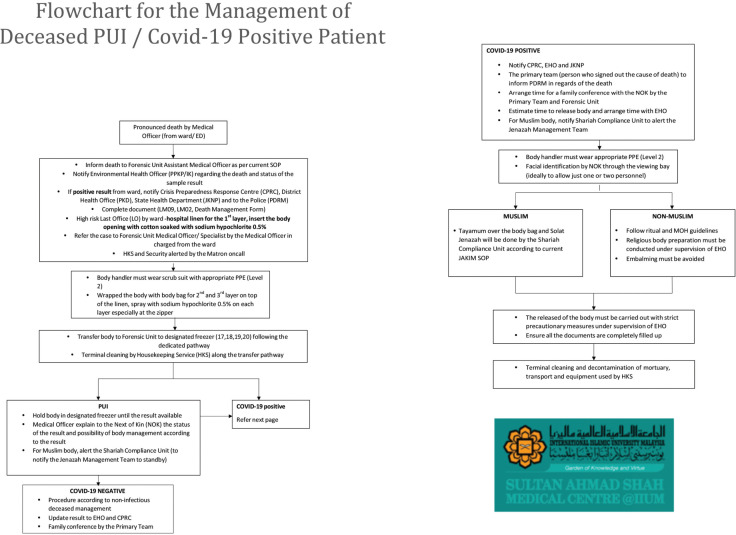
Flowchart for the management of deceased PUI / COVID-19 positive patients at Sultan Ahmad Shah Medical Centre @IIUM.

As the number of cases started to plummet, we develop a protocol was developed to resume elective surgeries and clinics while trying to minimise the risk of local transmission among HCWs and the patients (Fig. 2 to 9). During this period, the priority was to clear the backlog of surgical cases accumulated during the initial MCO period while minimising the risk of the patients contracting COVID-19 during their stay in the ward. During the period termed as the RMCO (Recovery Movement Control Order) which lasted from 10th June 2020 to 31st March 2021, we managed to perform elective surgeries such as joint replacement surgeries and tumour resection procedures. Meanwhile, we continued serving as a proxy hospital that managed all traumatic cases that required surgical intervention, the protocol outlined in the flowchart below. One of the highlights of the protocol was the setting up of medical and surgical transit wards, where we isolate high-risk intervention, the protocol outlined in the patients were isolated prior to admission into the general medical and surgical wards. By setting up the transition wards, this shielded our in-patients from being exposed to new patients whose COVID-status were still unknown during admission.

## Transition into Hybrid Hospital

In April 2021, Malaysia encountered a spike of COVID-19 cases, with daily confirmed cases ranging from four to five digits. As the main public hospital treating COVID-19 in the state, HTAA was unable to cope with the surging number of patients, many of whom were in category four and five. This was when our university hospital transitioned into a hybrid hospital, where we treat both non-COVID-19 and COVID-19 patients were treated alike.

Similar to other hospitals dealing with COVID-19, two general wards and one ICU were dedicated to cater for patients with COVID-19. As the burden of treating COVID-19 started to take its toll from April 2021 onwards the number of elective surgeries were reduced and eventually stopped completely from July 2021, with only semi-emergency surgeries continued. During this period, Malaysia experienced the highest number of confirmed cases on a daily basis, with cases ranging from ten to twenty thousand daily, compared to a meagre number in the hundreds only at the turn of the year.

## Vaccine: The Light at the End of Tunnel

COVID-19 vaccination was initiated in Malaysia on 24th February 202119,20. The vaccination programme in our centre was divided into three phases. In the first phase, our priority was to inoculate the HCWs the centre. After the commencement of vaccination in March 2021, we managed to inoculate more than 95% of HCWs in our hospital within two months of the commencement of vaccination. All personnel were given two doses of Pfizer-BioNTech vaccine with two weeks apart. The vaccination was compulsory for all HCWs except those who had medial exemptions. A special appointment date was given to all high-risk recipients such as those with history of allergy or anaphylaxis, with the anaesthetists were on stand-by and all recipients will be monitored slightly longer than the recommended 15 minutes.

After the vaccination of the frontliners, the university hospital functioned as one of the main public vaccination centres for the locality during the second and third phase of the vaccination programme. The second phase was started on 1st of June 2021, when all high-risk patients were given the priority to receive the vaccination. Meanwhile, the third phase in 19th July 2021, when we offered vaccination for the general public^21^. Within the first month, more than 15,000 people had their inoculation in the centre with more than 1000 doses administered to vaccinees daily^21^. Based on data by Ministry of Health Malaysia, Malaysia achieved 20% (6.695 million) of fully vaccinated population by 30th July 2021, 40% (13.279 million) by 22nd August 2021 and 60% (19.720 million) by 23th September 202120. By December 2021, 78% (25.548 million) of the population were fully vaccinated^20^. Adolescent (age 12-17) immunisation was initiated on 8th September 2021 and by 10th November 2021, more than 80% of adolescents in the country were fully vaccinated^20^. It was all going well until the emergence of Omicron variant in December 2021. Thus far, the early reports indicated that a booster dose was going to be needed to confer protection against this new variant^22,23^.

## Impact on Orthopaedic Training and Examination

As a teaching hospital for both undergraduate and postgraduate students, the pandemic had forced a transition from conventional in-person meeting to a virtual one. In order to maintain social distancing, classes, meetings, presentations and census were all conducted online through applications such as Zoom and Google meet. Similar to our counterparts in University Malaya, clinical sessions with students were conducted with limited number of participants and live stream online for others to watch^24^. Although hiccups were encountered initially, mainly due to technical issues, but as time went by, we were getting better at conducting classes and meeting online. In fact, it has now become a norm for meetings and classes to be conducted online.

As the designated host university conducting post-graduate examination in the year 2020, we were, however, compelled to postpone the exit examination (Orthopaedic Specialty Committee, OSC Part 2) in May 2020, during the height of the COVID-19 pandemic in our country, as this strained our capacity to make the necessary logistic arrangements. Furthermore, the uncertainty at the beginning of the pandemic also made any future preparation not possible, ergo the OSC Part 2 was postponed to November 2020. In order to reduce the risk of COVID-19, the conjoint examination was decentralised and simultaneously conducted in three centres, namely University Malaya (UM) / University Kebangsaan Malaysia (UKM) in Kuala Lumpur, University Sains Malaysia (USM) in Kota Bharu and International Islamic University Malaysia (IIUM) in Kuantan. The same arrangement was repeated for the May 2021 examination. In November 2021, with COVID-19 cases controlled and the population achieving the threshold of vaccination status, the first centralised OSC Part 2 was conducted entirely in our centre without any drawback.

## Future Directions

With the coronavirus continuing to mutate and the emergence of the Omicron variant, it seems that the war with COVID-19 will be a drawn-out one. From the healthcare perspective, we can only continue to provide service adjusted on the severity of COVID-19 from time-to-time. Similarly, it is important to ramp up the service of booster dose vaccination to the HCWs and general population, as initial reports showed that this offers better protection compared to the initial two-doses of vaccination. For the time being, there must be consistence and persistence in following the standard operation protocol (SOP) to limit the spread of the virus. Concurrently, virtual meetings and classes will remain the preferred medium for teaching and learning purposes.

## Conclusion

As the pandemic is not going to be curbed in a short period of time, we have shared our experiences and hospital protocol so that other centres or countries who struggle with the COVID-19 containment can take a leaf out of our experiences. No one is truly safe until every-one is safe, hence it is hoped that the distribution and administration of vaccines can be accelerated worldwide so that this pandemic can be stymied.

## References

[1] Huang C, Wang Y, Li X, Ren L, Zhao J, Hu Y (2020). Clinical features of patients infected with 2019 novel coronavirus in Wuhan, China.. Lancet..

[2] Zainol Rashid Z, Othman SN, Abdul Samat MN, Ali UK, Wong KK (2020). Diagnostic performance of COVID-19 serology assays.. Malays J Pathol..

[3] Zhang YZ (2020). Novel 2019 coronavirus genome.. Virological.

[4] Malik YA (2020). Properties of Coronavirus and SARS-CoV-2.. Malays J Pathol..

[5] Tay K, Kamarul T, Lok WY, Mansor M, Li X, Wong J (2020). COVID-19 in Singapore and Malaysia: Rising to the Challenges of Orthopaedic Practice in an Evolving Pandemic.. Malays Orthop J..

[6] World Health Organization (WHO) (2020). WHO Director-General's opening remarks at the media briefing on COVID-19 - 11 March 2020.

[7] Cucinotta D, Vanelli M (2020). WHO Declares COVID-19 a Pandemic.. Acta Biomed..

[8] World Health Organization (WHO) (2021). WHO Coronavirus (COVID-19) Dashboard.

[9] Che Mat NF, Edinur HA, Abdul Razab MKA, Safuan S (2020). A single mass gathering resulted in massive transmission of COVID-19 infections in Malaysia with further international spread. J Travel Med..

[10] World Health Organization (WHO) (2020). Coronavirus disease 2019 (COVID-19) situation report.

[11] World Health Organization (WHO) (2021). Countries: Malaysia: The current COVID-19 situation.

[12] Li Q, Guan X, Wu P, Wang X, Lei Zhou, Tong Y (2020). Early Transmission Dynamics in Wuhan, China, of Novel Coronavirus-Infected Pneumonia.. N Engl J Med.

[13] Gandhi M, Yokoe DS, Havlir DV (2020). Asymptomatic Transmission, the Achilles' Heel of Current Strategies to Control Covid-19.. N Engl J Med..

[14] Wolfel R, Corman VM, Guggemos W, Seilmaier M, Zange S, Muller MA (2020). Virological assessment of hospitalized patients with COVID-2019.. Nature.

[15] Arons MM, Hatfield KM, Reddy SC, Kimball Anne, James A, Jacobs JR (2020). Presymptomatic SARS-CoV-2 Infections and Transmission in a Skilled Nursing Facility.. N Engl J Med..

[16] Sutton D, Fuchs K, D’Alton M, Goffman D (2020). Universal screening for SARS-CoV-2 in women admitted for delivery.. N Engl J Med..

[17] Day M (2020). Covid-19: four fifths of cases are asymptomatic, China figures indicate.. BMJ..

[18] Selangor Journal (2021). Chronology of MCO phases in the country.

[19] World Health Organization (WHO) (2021). Status of COVID-19 Vaccines within WHO EUL/PQ evaluation process.

[20] Ministry of Health Malaysia Vaccinations in Malaysia. [updated 2021 December 19; cited 2021 December 19].

[21] International Islamic University Malaysia.

[22] Mahase E (2021). Covid-19: Omicron and the need for boosters.. BMJ..

[23] Dolgin E (2021). Omicron is supercharging the COVID vaccine booster debate.. Nature..

[24] Shamsul SA, Cheng T, Abbas AA (2020). Hybrid Model for Clinical Post-Graduate Teaching in a University Hospital.. Malays Orthop J..

